# Practical Chemistry and Pharmacy

**Published:** 1895-09

**Authors:** 

**Affiliations:** LaGrange, Ga.


### PRACTICAL CHEMISTRY AND PHARMACY.

EDITED BY HENRY R. SLACK, M.D., PH.M., LAGRANGE, GA.

### GOOD TEST FOR SUGAR IN THE URINE.

In the *New York Medical Journal,* July 27, Dr. A. R. Elliott, of Chicago, announces a new method and says:

The ideal test for sugar will be one that is at once reliable and sensitive, recognizing with certainty minute quantities of sugar in the urine, and one that requires the employment of never more than a few dops of urine in its application. Such a one the following method, devised and employed by myself, has thus far proved to be. With great reliability it combines extreme delicacy, and requires the employment of a minimum quantity of urine.

The formulæ, for its preparation and the details of its application, are as follows:

Solution No. 1.

| | |
|---|---|
| Cupric sulphate (C. P.) | gr. xxvij. |
| Glycerin pure | ʒ ijj. |
| Distilled water | ʒ ijss. |
| Liquid potassæ | ad ℥ iv. |

Dissolve the cupric sulphate in the glycerin and distilled water. Gentle heat will facilitate the solution. When cold, add the liquor potassæ and mix thoroughly.

Solution No. 2 is a saturated solution of chemically pure tartaric acid in distilled water.

The solutions are quite stable and will keep indefinitely.

Into a test tube pour a drachm of the cupric-oxide solution and gently boil over a spirit flame. Then add two or three drops—not more—of the tartaric-acid solution and boil again. Now add the suspected urine slowly, drop by drop, boiling and shaking the test solution between each drop until reduction takes place, or until eight drops of the urine have been added. If no change follows the addition of this amount of urine, sugar is not present. The end reaction is a yellowish or reddish, or sometimes greenish-gray deposit of suboxide which is marked and unmistakable. If the solution be stood aside for a few moments the reaction deepens.

Applied in this manner, the test will detect less than one part in a thousand of urine, or one tenth per cent. If sugar be present to any considerable extent, a single drop of urine will promptly develop the reaction. The addition of three drops gives a marked reduction when two grains to the ounce are present, and four drops will detect one grain to the ounce, or one in four hundred and eighty. More than eight drops of urine should never be used with this test, since that amount never fails to give a marked reaction when half a grain or more of sugar to the ounce is present, and smaller traces than this in the urine are of no interest to the practitioner.

---

### TO MAKE A GOOD EMULSION.

Two methods are employed in the manufacture of such emulsions. One consists in making a thick mucilage, to which the oil is added gradually in small proportions until it is all thoroughly incorporated, and lastly the other ingredients. The second method is, no doubt, universally employed in the leading pharmacies of this country, and its process is one in which most all druggists are well versed. Nevertheless, to accomplish a perfect success, I wish to suggest and impress a few very important points on this subject.

First of all, cleanliness, as in all other manipulations, is one of the agents which should never be lost sight of, and especially so in this case. A most convenient and advisable shape of mortar employed during this process is one of shallow form, with a flat pestle, properly adjusted to its shape. The powder, which should be of absolute purity, should be placed in the mortar, its dust covering the sides of the vessel, keeping the oil from greasing them. Now, the oil should be added in the proportion of one to two of the gum, and, after being well mixed, add a certain amount of water. Most any apprentice, after following these rules, should be able to turn out a first-class preparation. As it is, in these days of progress, druggists should provide themselves with an emulsifier, which would be a very useful machine to them, if they are in a community where emulsions are very frequently prescribed. A

perfect emulsion should be as white as milk, and its fat globules too small to be visible to the naked eye. In fact, it should be a homogeneous compound.—*Pacific Drug Review.*

---

### CLEANLINESS IN THE DRUG STORE.

Be clean in dispensing, as well as in other directions. Have the scale pans bright (ammonia and whiting, followed by Putzpomade, will do it). Rusty spatulas will spoil many a fine preparation; clean them with a piece of fine pumice stone slightly moistened with water. Don't chew the cork to make it fit the bottle; use a cork press always. Keep your graduates dry, clean, and in the rack. The majority of utensils used in dispensing should be kept in clean closets and drawers, out of the way and protected from dust. A drawer, however, is effective only when closed. It is dollars in your pocket to acquire the habit of closing every drawer or other container as soon as you have taken out what you want. The customer will notice it, too, and be impressed with your care and tidiness. Bottles for dispensing should always be dry, as well as clean. A drop of water will spoil the appearance of many a preparation; it renders oils, both fixed and volatile, very unsightly. And don't forget packages. Do them up carefully, with neatly folded corners and securely tied. An inside wrapper of oil or paraffin paper is the thing for ground flaxseed and other oily substances. And, then, the labels. Don't lick them in sight of the customer; have wet sponge handy, or a pastepot. An old knife kept at the sink and used to remove every old label will render unnecessary the caution never to paste a new label over an old one.

Be clean. There is but one thing more disgusting than a dirty drug store, and that is a dirty druggist.—*Ex.*

---

### CARBOLATE OF CAMPHOR.

Carbolate of camphor is prepared by adding one part by weight of carbolic acid to three parts of camphor, setting it aside for twenty-four hours, and straining through gauze. It is a permanent liquid, having a specific gravity of 990. It is thoroughly antiseptic, and possesses unsurpassed germicidal powers. It is used to impregnate gauze and cotton to be used as a dressing for wounds. It combines readily with alcohol, ether, fixed and essential oils, and petroleum derivations, but *not* with aqueous solutions, or *glycerin*. It readily dissolves menthol, cocaine, salicylic acid, iodoform, chloral hydrate, and mercuric chloride. When applied to inflamed or ulcerated mucous surfaces, it causes smarting for a moment, and then relieves existing pain, and acts as an antiseptic stimulant; mixed with an equal part of cotton seed oil, it forms a valuable dressing to incised, lacerated, or contused wounds, preventing suppuration. If applied after the suppurative process is established, it will change the character of the discharge, removing all fetor. In vaginitis, vulvitis, and pruritus vulvae, when applied to the vagina on cotton, which is kept in place for twenty-four hours, it relieves all distressing symptoms. After parturition a small quantity may be poured on the cloths that are applied to the vulva, to take up the discharges. It will remove all disagreeable fetor and relieve soreness, if ordinary cleanliness and proper changes are made. It is also a valuable application in cases of frost-bite. Finally, it may be used wherever an unirritating antiseptic is indicated.—*Southern Clinic.*

---

### SIMPLE TEST FOR SUGAR IN URINE.

Place 1 drachm (4 grammes) of urine in a test tube about one-half inch in diameter; add 1 drachm (4 grammes) of a saturated, aqueous solution of picric acid and ½ drachm (2 grammes) of liquor potassæ (P. B.). An orange-red color instantly appears, as a result of the incipient reducing action of creatinin upon picric acid at the ordinary temperature. If, after the liquid has been kept at the boiling point for about a minute, a bright-red color appears through the test tube when held up to the light, the urine may be confidently pronounced free from sugar. No other method as easy and rapid for clinical and life insurance purposes.—SIR GEORGE JOHNSON, *Lancet*, January 12, 1895.

